# Supplementary material for: Fatty Acids in Waste Tissues: The Nutraceutical Value of Gonads and Livers from the Moroccan Hypophthalmichthys molitrix and Cyprinus carpio Fishes
Source: Mar Drugs. 2023 Mar 17;21(3):188. doi: 10.3390/md21030188 (PMC10059766; doi:10.3390/md21030188)
Supplement: Supplementary file 1 [file marinedrugs-21-00188-s001.zip › marinedrugs-2237099-supplementary.pdf]

TABLE S1:

Table S1. Principal SFA, MUFA, and ω-6 FA (mean mg x100g^{-1} of tissue ± SE), of gonads, liver and fillet tissues of immature and mature common carp (*Cyprinus carpio*) and silver carp (*Hypophthalmichthys molitrix).*

	**SFA (mg/100g)**		**MUFA (mg/100g)**		**PUFA: ω-6^{f} FA (mg/100g)**	
	**C16:0 (mg/100g)**	**C18:0 (mg/100g)**	**C18:1 ω-9**	**C18:1 ω-7**	**C20:4ω-6**	**C18:2 ω-6,ω-9**
			Common carp immature			
**G**	21.59±1.18	6.51±0.04	24.39±1.46	5.79±0.29	14.36±1.02	8.94±0.32
**L**	117.59±6.72	56.60±0.89	93.09±2.74	44.75±2.52	nd.	14.56±2.31
**F**	10.77±5.52	6.32±3.52	11.80±6.52	4.21±2.67	4.63±1.82	3.48±2.30
			Silver carp immature			
**G**	18.94±3.81	7.48±1.25	37.51±11.14	5.80±1.19	4.21±0.65	13.71±3.96
**L**	55,28±22.53	15.33±5.25	22.19±7.67	9.23±3.58	nd.	9.33±5.00
**F**	5.41±1.74	4.82±1.87	4.59±1.62	1.17±0.50	1.68±0.69	0.92±0.44
			Common carp mature			
**G**	145.20 ±12.20	49.44±3.76	151.88±13.23	nd.	34.18±2.83	26.34±0.69
**L**	50.90±9.64	28.89±3.85	73.67±18.53	nd.	nd.	5.74±1.60
**F**	12.53±2.22	8.84±2.24	20.29±3.89	nd.	5.62±1.42	3.57±0.89
			Silver carp mature			
**G**	123.25±6.16	29.06±1.45	128.63±13.60	34,15±1,71	6.13±0.31	111.35±12.00
**L**	62.11±30.51	18.61±8.97	93.18±8.04	nd.	nd.	9.45±5.03
**F**	48.58±4.26	16.45±1.65	85.47±1.55	11,48±1,70	5.48±0.99	8.24±0.99

G: gonads tissues; **L**: liver tissues;**F**: fillet tissues: SFA in tissues , including C16:0 (Palmitic acid) and C18:0 (Stearic acid); MUFA in tissues, including, C18:1ω-7 (Vaccenic acid), C18:1ω–9 (Oleic acid); PUFA including, including C18:2ω-6 (Linoleic acid), C20:4ω-6 (Arachidonic acid).
